# The New Xpert MTB/RIF Ultra: Improving Detection of *Mycobacterium tuberculosis* and Resistance to Rifampin in an Assay Suitable for Point-of-Care Testing

**DOI:** 10.1128/mBio.00812-17

**Published:** 2017-08-29

**Authors:** Soumitesh Chakravorty, Ann Marie Simmons, Mazhgan Rowneki, Heta Parmar, Yuan Cao, Jamie Ryan, Padmapriya P. Banada, Srinidhi Deshpande, Shubhada Shenai, Alexander Gall, Jennifer Glass, Barry Krieswirth, Samuel G. Schumacher, Pamela Nabeta, Nestani Tukvadze, Camilla Rodrigues, Alena Skrahina, Elisa Tagliani, Daniela M. Cirillo, Amy Davidow, Claudia M. Denkinger, David Persing, Robert Kwiatkowski, Martin Jones, David Alland

**Affiliations:** a Department of Medicine, Rutgers New Jersey Medical School, Newark, New Jersey, USA; b Cepheid Inc., Sunnyvale, California, USA; c Cepheid Inc., Bothell, Washington, USA; d Public Health Research Institute, Rutgers, New Jersey Medical School, Newark, New Jersey, USA; e Foundation for Innovative New Diagnostics, Geneva, Switzerland; f National Center for Tuberculosis and Lung Diseases, Tbilisi, Georgia; g P. D. Hinduja National Hospital and Medical Research Centre, Mumbai, India; h National Reference Laboratory, Republican Scientific and Practical Centre for Pulmonology and Tuberculosis, Minsk, Belarus; i Emerging Bacterial Pathogens Unit, San Raffaele Scientific Institute, Milan, Italy; j Department of Preventive Medicine and Community Health, Rutgers, New Jersey Medical School, Newark, New Jersey, USA; Sequella, Inc.

**Keywords:** Xpert MTB/RIF Ultra, diagnosis, sensitive, tuberculosis

## Abstract

The Xpert MTB/RIF assay (Xpert) is a rapid test for tuberculosis (TB) and rifampin resistance (RIF-R) suitable for point-of-care testing. However, it has decreased sensitivity in smear-negative sputum, and false identification of RIF-R occasionally occurs. We developed the Xpert MTB/RIF Ultra assay (Ultra) to improve performance. Ultra and Xpert limits of detection (LOD), dynamic ranges, and RIF-R *rpoB* mutation detection were tested on *Mycobacterium tuberculosis* DNA or sputum samples spiked with known numbers of *M. tuberculosis* H37Rv or *Mycobacterium bovis* BCG CFU. Frozen and prospectively collected clinical samples from patients suspected of having TB, with and without culture-confirmed TB, were also tested. For *M. tuberculosis* H37Rv, the LOD was 15.6 CFU/ml of sputum for Ultra versus 112.6 CFU/ml of sputum for Xpert, and for *M. bovis* BCG, it was 143.4 CFU/ml of sputum for Ultra versus 344 CFU/ml of sputum for Xpert. Ultra resulted in no false-positive RIF-R specimens, while Xpert resulted in two false-positive RIF-R specimens. All RIF-R-associated *M. tuberculosis rpoB* mutations tested were identified by Ultra. Testing on clinical sputum samples, Ultra versus Xpert, resulted in an overall sensitivity of 87.5% (95% confidence interval [CI], 82.1, 91.7) versus 81.0% (95% CI, 74.9, 86.2) and a sensitivity on sputum smear-negative samples of 78.9% (95% CI, 70.0, 86.1) versus 66.1% (95% CI, 56.4, 74.9). Both tests had a specificity of 98.7% (95% CI, 93.0, 100), and both had comparable accuracies for detection of RIF-R in these samples. Ultra should significantly improve TB detection, especially in patients with paucibacillary disease, and may provide more-reliable RIF-R detection.

## IMPORTANCE

The Xpert MTB/RIF assay (Xpert), the first point-of-care assay for tuberculosis (TB), was endorsed by the World Health Organization in December 2010. Since then, 23 million Xpert tests have been procured in 130 countries. Although Xpert showed high overall sensitivity and specificity with pulmonary samples, its sensitivity has been lower with smear-negative pulmonary samples and extrapulmonary samples. In addition, the prediction of rifampin resistance (RIF-R) in paucibacillary samples and for a few *rpoB* mutations has resulted in both false-positive and false-negative results. The present study is the first demonstration of the design features and operational characteristics of an improved Xpert Ultra assay. This study also shows that the Ultra format overcomes many of the known shortcomings of Xpert. The new assay should significantly improve TB detection, especially in patients with paucibacillary disease, and provide more-reliable detection of RIF-R.

## INTRODUCTION

Tuberculosis (TB) remains a global health threat despite the development of new diagnostics and antitubercular drugs. The Xpert MTB/RIF assay (Xpert) was developed to improve TB and rifampin resistance (RIF-R) detection. This was accomplished by automating most of the steps required to process clinical samples and by improving the sensitive detection of both *Mycobacterium tuberculosis* and RIF-R. Xpert uses a hemi-nested PCR to amplify the rifampin resistance-determining region (RRDR) of the *M. tuberculosis rpoB* gene (1). Five *rpoB* RRDR-specific molecular beacons are then used to detect both the presence of *M. tuberculosis* and mutations responsible for approximately 95% of RIF-R. In initial analytical studies, Xpert detected *M. tuberculosis* directly from sputum, with a limit of detection (LOD) of 131 CFU/ml (1). The assay can be performed in approximately 2 h. Pooled data have shown Xpert to have an overall sensitivity and specificity of approximately 89% and 98%, respectively (2). Due to its ease of operation and large-scale applicability in settings outside conventional laboratories, Xpert substantially improved the diagnosis of RIF-R and multidrug-resistant (MDR) TB (2–4). Between its endorsement by the World Health Organization (WHO) in 2010 and 2016, over 23 million Xpert tests have been procured in 130 countries, resulting in a 3- to 8-fold increase in testing for MDR TB worldwide (3).

Xpert has been noted to have a number of limitations. Despite its excellent sensitivity in tests of smear-positive sputum samples, Xpert is somewhat less sensitive when testing smear-negative sputum. For example, when Xpert was performed following negative smear microscopy results, its pooled sensitivity was 67% (2). Xpert was 43% sensitive in one study of HIV-positive patients with smear-negative TB (5), and its sensitivity was as low as 28% for smear-negative TB patients from a high-resource country with a low TB incidence (6). Clinical impact has also been lower than originally expected, perhaps due to Xpert’s suboptimal negative predictive value, although Xpert did lead to more correct TB treatment overall (7). Assay sensitivity has also been limited in some types of extrapulmonary samples, which are known to contain lower levels of bacilli than pulmonary samples (8–10). Xpert has generally performed very well as a rapid test for RIF-R, with a pooled sensitivity and specificity of 94% and 98%, respectively (2). However, the assay was noted to have a limited capacity to detect RIF-R-associated mutations in mixed samples (11, 12), and in some reports, it has a decreased capacity to detect *rpoB* C533G mutations responsible for some cases of RIF-R (13). Xpert may also generate occasional false-positive RIF-R calls for paucibacillary samples due to delays in the real-time signal generated by assay probes D and E (14). Its false recognition of a nonfunctional *rpoB* F514F silent mutation as conferring RIF-R has also been reported (15).

Seeking to resolve these limitations associated with Xpert, we developed a next-generation assay for TB and RIF-R detection that we have termed the Xpert MTB/RIF Ultra assay (Ultra). Here, we describe the changes in the design of the cartridge, thermal-cycling parameters, PCR, and mutation detection chemistry and the resulting improvements in both TB detection and identification of RIF-R.

## RESULTS

### Sensitivity of Ultra versus that of Xpert.

**FIG 1:**
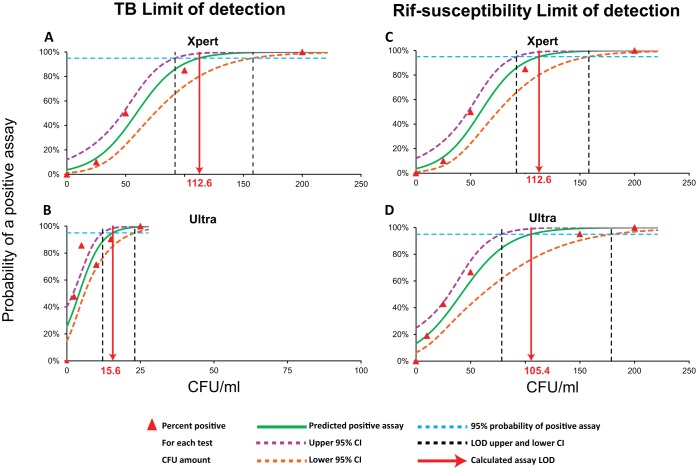
Limit of detection for *M. tuberculosis* H37Rv. The limit of detection of tuberculosis detection is shown for Xpert (A) versus Ultra (B). The limit of detection for generating a rifampin susceptibility rather than an indeterminate result is shown for Xpert (C) versus Ultra (D).

The comparative analytical LODs of Ultra and Xpert were tested by spiking serial dilutions of quantified CFU into sputum samples (Fig. 1). *M. tuberculosis* strain H37Rv contains 16 copies of IS*6110* and 5 copies of IS*1081*. For this test strain, Ultra and Xpert each detected the presence of both *M. tuberculosis* and RIF susceptibility correctly 100% of the time that a sample was tested, down to dilutions of 200 CFU/ml. At CFU inputs below 200, rates of correct TB-positive specimen detection by Xpert decreased to 85%, 50%, and 10% for 100, 50, and 25 CFU/ml, respectively. The calculated TB detection LOD for Xpert was 112.6 CFU/ml (95% confidence interval [CI], 91.9 to 158.2) (Fig. 1A). On the other hand, correct detection of TB-positive specimens by Ultra was 100% up to 25 CFU/ml. The calculated TB detection LOD for Ultra was 15.6 CFU/ml (95% CI, 12.2 to 23.1) (Fig. 1B), which represents an approximately 8-fold improvement over that of Xpert. The high sensitivity of Ultra was also evident at sub-LOD concentrations of added CFU, with 48% of the samples tested at 2.5 CFU/ml still positive by Ultra. Rates of detection of RIF susceptibility were comparable between Ultra and Xpert (LOD for RIF susceptibility by Xpert, 112.6 CFU/ml [95% CI, 91.9 to 158.2 CFU/ml] [Fig. 1C], versus LOD for RIF susceptibility by Ultra, 105.4 CFU/ml [95% CI, 78.5 to 178.8] [Fig. 1D]), although at sub-LOD concentrations, Ultra showed more positive results for RIF susceptibility than Xpert. Ultra also showed a substantial LOD improvement for detection of *M. bovis* BCG. In contrast to H37Rv, BCG contains only 1 copy of IS*6110* and 5 copies of IS*1081*. Even under these circumstances, the BCG detection limit for Xpert was 344.1 CFU/ml (95% CI, 297.5 to 434.0) (see Fig. S1A in the supplemental material), compared to a BCG detection limit for Ultra of 143.4 CFU/ml (95% CI, 106.2 to 243.7) (Fig. S1B). As was the case with the H37Rv *M. tuberculosis* strain, with BCG, the LODs of RIF susceptibility detection were similar by the two assays, with a small improvement evident by Ultra (LOD for RIF susceptibility in BCG by Xpert, 338.8 CFU/ml [95% CI, 294.0 to 425.8]; LOD for RIF susceptibility in BCG by Ultra, 300.3 CFU/ml [95% CI, 244.2 to 408.2]) (Fig. S1C and D).

10.1128/mBio.00812-17.2 FIG S1  Limit of detection for *M. bovis* BCG. The limit of detection of *M. bovis* BCG is shown for Xpert (A) versus Ultra (B). The limit of detection for generating a rifampin susceptibility rather than an indeterminate result is shown for Xpert (C) versus Ultra (D). Download FIG S1, EPS file, 2.4 MB. Copyright © 2017 Chakravorty et al. 2017 Chakravorty et al. This content is distributed under the terms of the Creative Commons Attribution 4.0 International license.

### Detection of mutations associated with rifampin resistance and silent mutations.

**FIG 2:**
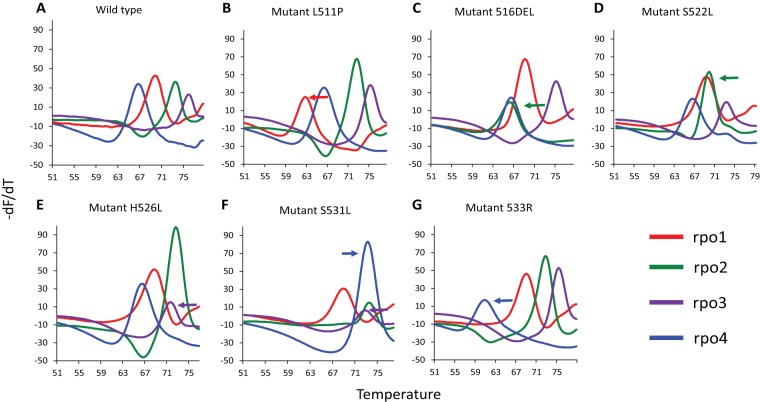
Detection of *M. tuberculosis rpoB* gene mutations associated with rifampin resistance. Derivative-transformed curves of four *rpoB*-specific sloppy molecular beacons (rpo1, rpo2, rpo3, and rpo4) first hybridized and then melted off of their *M. tuberculosis rpoB* gene amplicon target are shown. Each colored peak indicates the melting temperature of the probe corresponding to the colored line. The peaks for wild-type *M. tuberculosis* (A) identify a test sample as rifampin susceptible. The shift in one or more of the peaks away from the wild type’s melting temperature (B to G), identify a sample as an *rpoB* mutant and rifampin resistant. The shift in the melt peak is indicated by arrows.

We had shown previously that a combination of three *rpoB* sloppy molecular beacon (SMB) probes could be used in an assay to detect and differentiate *M. tuberculosis* RRDR mutations associated with RIF-R (16). For Ultra, we further refined a set of *rpoB* SMBs so that detection of RRDR codon 533 mutants associated with RIF-R was improved (particularly when the SMB probes were mixed with wild-type DNA). We also modified one of the *rpoB* SMBs so that it specifically differentiated the silent mutations Q513Q and F514F from other RRDR mutations that were associated with RIF-R. To improve detection of RRDR codon 533 mutants, we added an additional mutation-detecting *rpoB* SMB probe (rpo4), thereby expanding the *rpoB* SMB set from three to four. The rpo4 SMB was also designed to be more complementary to *rpoB* S531L mutants than to the wild-type sequence, which resulted in an increased melting temperature (*T*_*m*_) in the presence of this mutation compared to the wild type’s *T*_*m*_. To distinguish the silent mutations from RIF-R-associated mutations, we redesigned the rpo1 SMB probe so that its *T*_*m*_ increased upon hybridizing to Q513Q and F514F mutants compared to the *T*_*m*_ generated upon hybridizing to the wild-type sequence. This SMB probe continued to generate *T*_*m*_ values less than the wild type’s *T*_*m*_ when it hybridized to RRDR mutants associated with RIF-R. We challenged Ultra with 25 different RRDR mutations covering almost the entire *rpoB* RRDR from codons 510 to 533. The panel also included double and triple mutations, deletions, and stop codons and the Q513Q and F514F silent mutations (Table 1). The results showed that all mutations resulted in a reproducible and measurable shift in melting *T*_*m*_ peaks by one or more of the *rpoB* SMB probes (Fig. 2). Furthermore, each RIF-R-conferring mutation was reproducibly identified by the GeneXpert software as “RIF resistance detected,” except with a triple mutant that was identified as “RIF indeterminate” due to a *T*_*m*_ that was below the software’s mutation detection window (Table 1). In contrast, the two silent mutations Q513Q and F514F were identified as “RIF resistance not detected.” Xpert has been known to occasionally produce false RIF-R results in paucibacillary samples (14). However, we did not observe false RIF-R in this sample type using Ultra. Examining all Ultra runs performed on sputum spiked with ≤200 CFU/ml over a period of several months, 0/164 (0%) of the assays with a RIF susceptibility result were falsely resistant. In contrast, of all Xpert runs performed on sputum spiked with ≤200 CFU/ml over a similar period, 2/74 (2.7%) of the assays with a RIF susceptibility result were falsely positive.

**TABLE 1:** Ultra mutation panel challenge

Mutation(s)	Probe(s) for RIF-R detection	Ultra “test result” (semi-quantitation and RIF-R calls)
None		MTB DETECTED MEDIUM;RIF Resistance NOT DETECTED
Q510V + D516Y	rpo1, rpo2	MTB DETECTED MEDIUM;RIF Resistance DETECTED
L511P	rpo1	MTB DETECTED MEDIUM;RIF Resistance DETECTED
512T + M515I + H526N	rpo1, rpo2, rpo3	MTB DETECTED MEDIUM;RIF Resistance INDETERMINATE
Q513K	rpo1	MTB DETECTED MEDIUM;RIF Resistance DETECTED
Q513Q (silent)		MTB DETECTED MEDIUM;RIF Resistance NOT DETECTED
F514F (silent)		MTB DETECTED HIGH;RIF Resistance NOT DETECTED
516DEL	rpo2	MTB DETECTED MEDIUM;RIF Resistance DETECTED
D516V	rpo2	MTB DETECTED MEDIUM;RIF Resistance DETECTED
518DEL	rpo2	MTB DETECTED MEDIUM;RIF Resistance DETECTED
S522L	rpo2, rpo3	MTB DETECTED MEDIUM;RIF Resistance DETECTED
H526C	rpo3	MTB DETECTED HIGH;RIF Resistance DETECTED
H526D	rpo3	MTB DETECTED MEDIUM;RIF Resistance DETECTED
H526L	rpo3	MTB DETECTED MEDIUM;RIF Resistance DETECTED
H526N	rpo3	MTB DETECTED LOW;RIF Resistance DETECTED
H526R	rpo3, rpo4	MTB DETECTED HIGH;RIF Resistance DETECTED
H526Y	rpo3	MTB DETECTED MEDIUM;RIF Resistance DETECTED
526Stop	rpo4	MTB DETECTED HIGH;RIF Resistance DETECTED
R529K	rpo4	MTB DETECTED MEDIUM;RIF Resistance DETECTED
S531L	rpo4	MTB DETECTED MEDIUM;RIF Resistance DETECTED
S531Q	rpo4	MTB DETECTED MEDIUM;RIF Resistance DETECTED
S531W	rpo4	MTB DETECTED MEDIUM;RIF Resistance DETECTED
L533P	rpo4	MTB DETECTED MEDIUM;RIF Resistance DETECTED
L533R	rpo4	MTB DETECTED MEDIUM;RIF Resistance DETECTED
L533P + H526S	rpo3, rpo4	MTB DETECTED MEDIUM;RIF Resistance DETECTED
L530M + S531P	rpo4	MTB DETECTED MEDIUM;RIF Resistance DETECTED

### Detection of heteroresistance.

**FIG 3:**
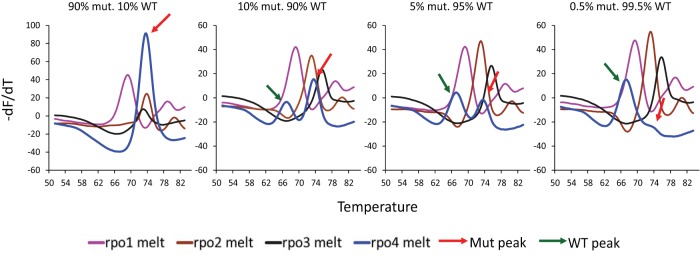
Detection of hetero-resistance. Samples containing equal quantities of total *M. tuberculosis* DNA were created using different proportions of wild-type and rifampin-resistant *rpoB* S531L mutant DNA. Derivative-transformed curves of four *rpoB*-specific sloppy molecular beacons (rpo1, rpo2, rpo3, and rpo4) first hybridized and then melted off of their *M. tuberculosis rpoB* gene amplicon target are shown. The presence of rifampin-resistant mutant DNA, as indicated by the peak marked with a red arrow, was consistently detected in samples containing as little as 10% mutant DNA and in a subset of samples containing as little as 5% mutant DNA. WT, wild type; mut., mutant.

We assessed the ability of Ultra to detect heteroresistance by testing mixtures of wild-type DNA and DNA containing the *rpoB* S531L mutation, which is the most commonly occurring of the RRDR mutations (16). The rpo4 SMB probe was designed to generate a higher *T*_*m*_ value in the presence of the S531L mutation than that of the wild type. This probe design resulted in a predominating mutant peak in mixtures containing as little as 10% mutant DNA and a distinct double peak in a proportion of mixtures containing as little as 5% mutant DNA (Fig. 3), resulting in a GeneXpert output of “RIF resistance detected.” The assay failed intermittently to detect RIF-R in mixtures containing 5% mutant samples and reproducibly failed to detect this mutation only when the mutant DNA was present at ≤1% (Fig. 3). Heteroresistance detection for other RRDR mutations was not as sensitive as it was for the S531L mutation because these other mutations were detected by a downward *T*_*m*_ shift. However, even for the L511P and H526N RRDR mutations tested, RRDR mutations in mixtures containing *rpoB* mutant DNA at levels between 20% and 40% could still be reliably detected.

### Assay inclusivity.

We examined the capacity of Ultra to detect a wide variety of *M. tuberculosis* strains with varied numbers or no copies of IS*6110*. DNAs from 22 different clinical *M. tuberculosis* isolates and from one strain each of *Mycobacterium bovis*, *M. bovis* BCG, and *M. tuberculosis* H37Rv containing 0 to 18 copies of IS*6110* (Table S1), representing 5 different genotypic lineages (17), were tested at a DNA input of approximately 4 times the estimated LOD of Ultra. The presence of *M. tuberculosis* was detected in all four replicates of each of the 25 different DNA samples (Table S1), indicating broad inclusivity.

10.1128/mBio.00812-17.6 TABLE S1  Inclusivity testing results for Ultra. Download TABLE S1, DOCX file, 0.01 MB. Copyright © 2017 Chakravorty et al. 2017 Chakravorty et al. This content is distributed under the terms of the Creative Commons Attribution 4.0 International license.

### Analytical specificity and exclusivity.

The analytical specificity of the assay was tested on 30 different isolates of nontuberculous mycobacteria (NTM) and 18 different Gram-positive and -negative bacteria in triplicate samples to assess the cross-reactivity of the assay primers and probes. No signals were generated from the two *M. tuberculosis* detection probes targeting the IS*6110* and IS*1081* genes, with a result output of “MTB not detected” (where “MTB” stands for *M. tuberculosis*) for all the replicates tested. We observed weak rpo2 probe signals (average cycle threshold [*C*_*T*_] value of 30 or greater) for most of the NTM and additional rpo1 probe signals (average *C*_*T*_ of 36) for one replicate each of *Mycobacterium asiaticum*, *Mycobacterium interjectum*, and *Mycobacterium shimoidei*. However, these late signals from mostly a single *rpoB* probe did not lead to false *M. tuberculosis* or RIF-R detection, since none of these samples produced signals from the *M. tuberculosis*-specific probes and the assay does not proceed to melt analysis and RIF-R detection in the absence of an *M. tuberculosis*-specific signal. Additionally, we tested for the possibility that NTM might act as an interfering substance, which might confound TB detection in cases of mixed infection with both *M. tuberculosis* and an NTM. We mixed 10^6^ CFU/ml of the clinically relevant NTM, namely, *Mycobacterium abscessus*, *Mycobacterium avium*, *Mycobacterium intracellulare*, and *Mycobacterium kansasii* or the environmental contaminant *Mycobacterium gordonae*, with 50 CFU/ml of *M. tuberculosis*. Each of these mixtures was tested in triplicate. All tests showed the presence of RIF-susceptible TB, indicating that high background levels of NTM do not significantly interfere with the performance of Ultra for TB detection.

### Dynamic range and semiquantitative measurements compared to those of Xpert.

**FIG 4:**
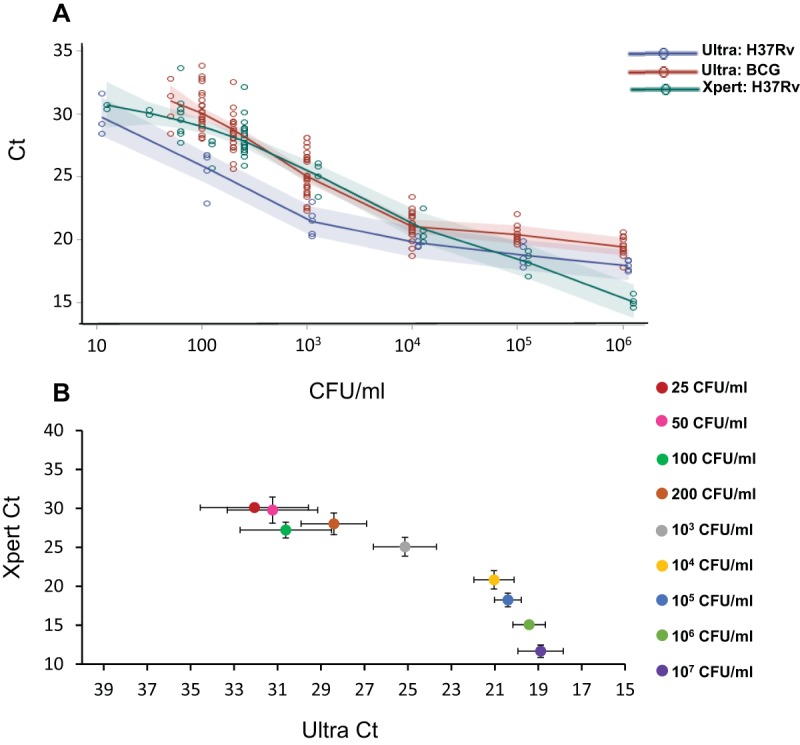
Comparative dynamic range of Ultra and Xpert. Log dilutions of *M. tuberculosis* H37Rv spiked into sputum samples (minimum of 20 samples per dilution) were tested by Ultra and Xpert. The same dilutions of BCG were also tested by Ultra. (A) The *C*_*T*_ values for each CFU dilution are shown. Colored circles show the *C*_*T*_ of each positive result. Assay results that were negative are not shown, as these did not result in any *C*_*T*_ value. Colored shading shows ± 1 standard deviation. (B) The experimental results for the H37Rv dynamic-range study comparing the mean *C*_*T*_ values of each log dilution produced by Ultra versus Xpert are also shown. Error bars show ± 1 standard deviation.

We performed repeated experiments with 10-fold serial dilutions of *M. tuberculosis* H37Rv and *M. bovis* BCG ranging from 10^7^ CFU/ml to 10 CFU/ml spiked in sputum to evaluate the dynamic range of Ultra. These results were compared to those generated by Xpert in tests of the same spiked samples (Fig. 4A). We used the lowest *C*_*T*_ generated among the four rpo probes during the nested-PCR stage of Ultra (or of the five *rpoB* probes of Xpert) as a semiquantitative measure of the *M. tuberculosis* cell number in each test sample. This semiquantitative measure is similar to that performed during routine clinical use of Xpert. In Ultra, sputum samples spiked with 10^6^ and 10^5^ CFU/ml of *M. tuberculosis* H37Rv generated similar average *rpoB C*_*T*_ values of approximately 19 to 20. Samples spiked with 10^4^ CFU/ml averaged only slightly higher *rpoB C*_*T*_ values of 20 to 21. From 10^4^ to 10 CFU/ml, average *rpoB C*_*T*_ values showed a linear relationship to CFU numbers (Fig. 4A). A similar trend was observed in sputum samples spiked with BCG, although the *rpoB C*_*T*_ values were slightly higher for each CFU. The relationship between the *rpoB C*_*T*_ value and input CFU also allowed us to classify samples into “high,” “medium," “low,” and “very low” categories (Fig. S2). Most of the samples containing 100 CFU/ml were *rpoB* probe positive, resulting in “low” or “very low” result outputs. However, at CFU inputs below 100 CFU/ml, particularly in the range of 10 CFU/ml, the number of positive TB assays (i.e., IS*6110*/IS*1081* positive) which also resulted in positive *rpoB* probe signals decreased to 20% or less, resulting in fewer *M. tuberculosis*-positive assays being associated with an *rpoB C*_*T*_ that could be used in semiquantitation. We introduced the “trace” category to identify the paucibacillary samples which were IS*6110*/IS*1081* positive but *rpoB* negative. Ultra tests of BCG dilutions behaved similarly, although the dynamic range curve appeared to flatten out at slightly higher numbers of CFU per milliliter for both H37Rv and BCG (Fig. 4A). In comparison, Xpert showed a better linear trend of the *rpoB C*_*T*_ versus CFU input than did Ultra over the entire dynamic range, particularly at the higher concentrations of 10^7^ to 10^5^ CFU/ml, as shown for H37Rv in Fig. 4A. By definition, Xpert did not result in any tests that fell into the positive semiquantitative “trace” category. The altered relationship between the Ultra *C*_*T*_ and the Xpert *C*_*T*_ in samples with increasing numbers of CFU was even more apparent when Ultra and Xpert *C*_*T*_ results were plotted against each other at each CFU dilution (Fig. 4B). For semiquantitative outputs, the following *rpoB C*_*T*_ values were chosen for Ultra. *C*_*T*_ values of 15 to 18.9 indicated “high,” 19 to 24.9 indicated “medium,” 25 to 28.9 indicated “low,” and 29 to 40 indicated “very low” levels of bacteria (Fig. S2). Together, these results show that the redesign of Ultra decreased the semiquantitative capacity at the high end of numbers of CFU per milliliter but retained this capacity in more paucibacillary samples corresponding to “+1,” “scanty,” and “smear negative.”

10.1128/mBio.00812-17.3 FIG S2  Semiquantitative estimation of bacterial load using Ultra. Log dilutions of *M. tuberculosis* H37Rv and *M. bovis* BCG spiked into sputum samples (minimum of 20 samples per dilution) were tested by Ultra. The earliest *rpoB C*_*T*_ values for each CFU dilution for both BCG and H37Rv are shown. Colored circles show the mean earliest *rpoB C*_*T*_ of each positive result, with the shaded areas showing the semiquantitative measure of the bacterial load in the sample from “high” to “very low.” Spiked samples below the LOD of the *rpoB* assay, which resulted in no *rpoB C*_*T*_ but only a FAM *C*_*T*_ (IS*6110*/IS*1081*) were designated “trace,” with colored triangles representing the “zero” *rpoB C*_*T*_s. Download FIG S2, EPS file, 1.2 MB. Copyright © 2017 Chakravorty et al. 2017 Chakravorty et al. This content is distributed under the terms of the Creative Commons Attribution 4.0 International license.

### Comparative performances of Ultra and Xpert on clinical sputum samples.

We performed a limited, retrospective evaluation of the performances of Ultra and Xpert using 277 sputum samples (Table 2). Compared to culture results, the overall sensitivity and specificity of Ultra for TB detection were 87.5% (95% CI, 82.1, 91.7) and 98.7% (95% CI, 93.0, 100%), respectively, and those of Xpert were 81.0% (95% CI, 74.9, 86.2) and 98.7% (95% CI, 93.0, 100%), respectively. The sensitivity of Ultra with the 109 smear negative-culture positive samples was 78.9% (95% CI, 70.0, 86.1) compared to 66.1% (95% CI, 56.4, 74.9) for Xpert, resulting in an approximately 13%-increased sensitivity for Ultra. For smear-positive and culture-positive samples, the sensitivities of Ultra and Xpert were 97.8% (95% CI, 92.3, 99.7) and 98.9% (95% CI, 94.0, 100), respectively. RIF susceptibility results were available for 187/200 culture-positive samples. The sensitivity and specificity of Ultra for the detection of RIF susceptibility were 92.7% (95% CI, 80.1, 98.5) and 98.0% (95% CI, 92.8, 99.9), respectively, while that of Xpert were 92.7% (95% CI, 80.1, 98.5) and 99.0% (95% CI, 94.4, 100), respectively. One sample was detected as RIF resistant by Ultra; it was susceptible by both phenotypic susceptibility testing and Xpert. Upon Sanger sequencing, this sample was found to be hetero-resistant, with a mixture of the wild type and S531L mutant, and was detected as resistant only by Ultra and missed by both the RIF susceptibility test and Xpert.

**TABLE 2:** Comparative performance of Ultra and Xpert assay on clinical sputum samples

Assay	Tuberculosis detection			Rifampin resistance detection	
	% sensitivity (95% CI)		% specificity (95% CI) (*n =* 77)	% sensitivity (95% CI) (*n =* 41)	% specificity (95% CI) (*n =* 98)
	All culture-positive specimens (*n =* 200)	Smear-negative specimens (*n =* 109)			
Xpert	81.0 (74.9, 86.2)	66.1 (56.4, 74.9)	98.7 (93.0, 100)	92.7 (80.1, 98.5)	99.0 (94.4, 100)
Ultra	87.5 (82.1, 91.7)	78.9 (70.0, 86.1)	98.7 (93.0, 100)	92.7 (80.1, 98.5)	98.0^a^ (92.8, 99.9)

^a^ One sample was detected as RIF resistant by Ultra but was detected as RIF susceptible by both phenotypic susceptibility testing and Xpert. Upon Sanger sequencing, this sample was found to be hetero-resistant with a mixture of the wild type and an *rpoB* S531L mutant. Reclassifying this sample as RIF resistant would change the sensitivity and specificity for RIF-R detection for Ultra to 92.9% (95% CI, 80.5, 98.5) and 99.0% (95% CI, 94.4, 100), respectively, and would change the sensitivity and specificity for RIF-R for Xpert to 90.5% (95% CI, 77.4, 97.3) and 99.0% (95% CI, 94.4, 100), respectively. Note that Ultra provided RIF resistance results for 20 samples that tested *M. tuberculosis* negative by Xpert and are excluded from the analysis of RIF resistance to provide accuracy estimates on the same sample set. Xpert was performed on a fresh sputum aliquot, and Ultra was tested retrospectively on a frozen aliquot of the same sputum sample.

## DISCUSSION

We have developed an advanced version of the Xpert MTB/RIF assay with better TB detection capabilities and more definitive identification of RIF susceptibility and resistance. The (i) inclusion of two new PCR assays that target two different multicopy genes, (ii) conversion of the *rpoB* and IS*6110* assays into fully nested PCRs, and (iii) use of a larger PCR tube that doubles the amount of sample DNA that can be tested have allowed for an almost 10-fold increase in analytical sensitivity for the detection of *M. tuberculosis* H37Rv. Importantly, this increase in sensitivity was not restricted only to H37Rv, which contains 16 copies of the IS*6110* assay target. Sensitivity was also more than doubled for the detection of BCG, which contains only one IS*6110* copy, suggesting that the additional sensitivity gained by Ultra is expected to be true for different clinical *M. tuberculosis* strains containing both high and low numbers of copies of IS*6110*. A limited study on frozen clinical samples which demonstrated improved sensitivity for Ultra compared to that of Xpert supports this conclusion. The inclusion of the IS*1081* reaction also ensures that the assay will detect rare *M. tuberculosis* strains that do not contain any IS*6110* genes (18), as shown by our analytical experiments that included a zero-copy-number IS*6110* strain.

Ultra also enables improved detection of RRDR mutations associated with RIF-R. Both Xpert and Ultra detect RIF-R when one or more of the *rpoB*-specific molecular beacons used in the assays exhibit altered binding to an RRDR mutant DNA target. In Xpert, there is a *C*_*T*_ delay in the binding of one the molecular beacons to the mutant RRDR sequence relative to the binding of the remaining molecular beacons to the wild-type sequence. A delay (delta *C*_*T*_) of >4 cycles defines RIF-R. Unfortunately, this approach mistakenly identifies silent mutations as causes of RIF-R (15). Xpert also has a limited ability to identify hetero-resistance (11, 19), and small amounts of target DNA (as may occur in paucibacillary samples) can produce a *C*_*T*_ delay which mimics a RIF-R hybridization pattern (14). We observed such false RIF-R calls from Xpert in our analytical experiments when low concentrations of *M. tuberculosis* and BCG CFU were tested; however, these falsely resistant calls were completely absent in the Ultra runs. The replacement of the delta *C*_*T*_ approach for mutation detection with the delta *T*_*m*_ approach (16) used in Ultra enabled us to resolve these limitations, producing a more robust assay. An additional benefit of the delta *T*_*m*_ approach is that the many RRDR mutations can also be specifically identified by their *T*_*m*_ values (16).

The enhanced sensitivity of Ultra allowed us to identify more cases of smear-negative and culture-positive TB and more TB cases overall when Ultra was used on 277 sputum samples from TB suspects. Levels of RIF resistance detection were comparable between Xpert and Ultra, although Ultra detected a hetero-resistant sample that was missed by both phenotypic susceptibility testing and Xpert. Our analytic results have confirmed that Ultra has an improved ability to detect resistance in mixed samples, an observation that likely explains why Ultra detected RIF-R in the hetero-resistant clinical sample. Importantly, the manual steps required to perform Xpert and Ultra are identical. Furthermore, the two assays can be run in identical GeneXpert instruments after a software upgrade. Thus, it is expected that Ultra can be implemented with little additional training in sites that already use the current Xpert assay.

The increase in sensitivity provided by Ultra is not without certain risks. Increased sensitivity may predispose to false-positive results due to sample cross contamination, especially in laboratories that process numerous samples from TB suspects and *M. tuberculosis* cultures. Furthermore, increased false-positive results have been noted when Xpert was used to test subjects with recent a history of TB (20). It is possible that Ultra will be even more predisposed to false-positive results in this situation. In this regard, our limited study on stored samples was performed at some sites with a relatively low population-level incidence of TB, and the specificity decrements for Ultra may be greater if the assay is implemented in high-TB-incidence countries. A prospective multicenter study will soon report on the clinical performance of Ultra compared to Xpert in a more representative TB population (21).

The Ultra assay analysis algorithm is designed to report a RIF-R result as indeterminate in most situations where ≥1 of the *rpoB* SMBs do not produce any measurable *T*_*m*_ peak. This was done to protect the assay from false-positive RIF-R calls in the rare (hypothetical) circumstance that a test performed below the LOD for RIF-R might produce >1 but <4 *T*_*m*_ peaks. The consequence of this algorithm is that large RRDR deletions or multiple mutations might destabilize one or more of the *rpoB* SMB probes sufficiently to eliminate any *T*_*m*_ in the temperature range measured by the assay. This might produce indeterminate RIF-R results in rare samples that are actually RRDR RIF-R mutants, as observed with a triple mutant in our analytical testing experiments (Table 1).

Our results suggest that Ultra will result in greater TB case detection rates not only in subjects with paucibacillary TB, such as those with HIV coinfection, but also in pediatric patients with TB and those with extrapulmonary TB, which are known to have lower mycobacterial loads. As TB elimination programs achieve initial successes, rates of paucibacillary TB should increase compared to rates of smear-positive disease. In this setting, assays with increased sensitivity, such as Ultra, may be instrumental in identifying the remaining cases of TB, and these assays are likely to prove valuable in furthering WHO goals to eradicate this disease.

## MATERIALS AND METHODS

### Human-subject approvals.

This study was approved by the University of Medicine and Dentistry of New Jersey (now Rutgers University) Institutional Review Board (IRB; protocol numbers 020160000657 and 0120090104), by the ethics committee of the National Center for Tuberculosis and Lung Diseases of Georgia (protocol numbers IRB00007705 and IORG0006411), and by the ethics committee of P. D. Hinduja Hospital and Medical Research Center (IRB research protocol number 954-15-CR).

### Cartridge configuration and assay composition.

The Ultra assay cartridge is a modified version of the G4 cartridge used in Xpert (Cepheid, Sunnyvale, CA, USA). It consists of a multiposition fluidic valve, a bacterial-capture filter, and 11 chambers containing buffers needed for sample processing and PCR as described previously (1), and the integrated 25-µl capacity PCR tube was redesigned to have a 50-µl volume capacity. The primers and molecular beacon or TaqMan probes used in the assay were also changed relative to those of Xpert (see Tables S2 and S3 in the supplemental material). Ultra uses four relatively long sloppy molecular beacon (SMB) probes that together target the *M. tuberculosis* RRDR to detect RIF-R, a function originally performed by five conventional molecular beacons targeted to the RRDR in Xpert (1). The alignment of the Xpert and Ultra probes targeting the *rpoB* RRDR is shown in Fig. S3. Two additional probes, a TaqMan probe that targets the *M. tuberculosis* multicopy IS*6110* gene and a molecular beacon that targets the multicopy IS*1081* gene, substitute for the *rpoB* molecular beacons used in Xpert to perform the task of *M. tuberculosis* detection. Each *rpoB*-specific SMB probe is labeled with a different fluorophore, allowing them to be individually analyzed. However, both the IS*6110*- and IS*1081*-specific probes are labeled with 6-carboxyfluorescein (FAM). As a consequence, a merged FAM signal generated by the combined signal of both FAM-labeled IS*6110* and IS*1081*-specific probes is used to identify the presence of *M. tuberculosis*. The assay microfluidics of Ultra were modified from those of Xpert to add more volume of processed DNA to the PCR tube, and a faster PCR program was developed to enable an earlier time to result. The complete details of the cartridge configuration and reaction components are described fully in the supplemental material.

10.1128/mBio.00812-17.7 TABLE S2  Sequences of the primers used in Ultra. Download TABLE S2, DOCX file, 0.02 MB. Copyright © 2017 Chakravorty et al. 2017 Chakravorty et al. This content is distributed under the terms of the Creative Commons Attribution 4.0 International license.

10.1128/mBio.00812-17.8 TABLE S3  Sequences of the probes used in Ultra. Download TABLE S3, DOCX file, 0.02 MB. Copyright © 2017 Chakravorty et al. 2017 Chakravorty et al. This content is distributed under the terms of the Creative Commons Attribution 4.0 International license.

10.1128/mBio.00812-17.4 FIG S3  Alignment of Ultra and Xpert probes along the *M. tuberculosis rpoB* RRDR. Probe sequence map showing an overlay of the probes used to target the *rpoB* RRDR mutations in Ultra and Xpert. rpo1 to 4: Ultra *rpoB* probes; probes A to E, Xpert *rpoB* probes; uppercase letters, probe sequences that are complementary to the target; black lowercase letters, probe stems; red lowercase letters, mutations inserted in the probes; U, deoxy-uracil; MTB^1^, *Mycobacterium tuberculosis*. Download FIG S3, EPS file, 1 MB. Copyright © 2017 Chakravorty et al. 2017 Chakravorty et al. This content is distributed under the terms of the Creative Commons Attribution 4.0 International license.

### Ultra procedure.

The sample treatment and cartridge loading processes used were the same as described previously for Xpert (1). Briefly, each sample (clinical sample, spiked sputum, or cultured *M. tuberculosis* CFU) was first mixed at a 2:1 ratio with a commercial NaOH- and isopropanol-containing sample reagent (SR; Cepheid, Sunnyvale, CA). The mixture was incubated for 15 min with occasional shaking and then added to the sample loading chamber of the cartridge for automatic processing as described fully in the supplemental material. The *M. tuberculosis* and RIF-R detection algorithm is shown in Fig. S4. The presence of *M. tuberculosis* was detected by the real-time signal from the probes targeting the multicopy IS*6110* and IS*1081* genes (22–24). The *T*_*m*_ values generated by the *rpoB* SMB probes were used to identify the presence of either the wild-type or the mutant RRDR DNA sequence. The cycle threshold (*C*_*T*_) of the first positive *rpoB* probe was also used for Ultra’s semiquantitative function. The semiquantitative categories of high, medium, low, and very low were similar to those of Xpert. An additional semiquantitative category of “trace” was introduced in Ultra. The trace result identified samples that were *M. tuberculosis* positive due to the presence of the IS*6110* and/or IS*1081* molecular signals (*C*_*T*_ ≤ 37) in the absence of a signal from at least 3 of the *rpoB* SMBs. The “trace” category was designed to identify samples with the lowest number of *M. tuberculosis* targets. Automated TB detection and RIF susceptibility calls were performed by modified GeneXpert Diagnostics software (Cepheid, Sunnyvale, CA). Real-time PCR signals obtained during the second nested-amplification phase indicated the absence or presence of *M. tuberculosis*, which was followed by a post-PCR melting temperature (*T*_*m*_) analysis only if the positive sample was not identified as “trace.” The *T*_*m*_ values generated from the *rpoB* SMB probes were then used to perform a RIF susceptibility call. The result outputs from the automated assay software were labeled as follows. (i) In the absence of any real-time signal other than the PCR internal control (IC), the result was “MTB not detected” (the assay did not proceed to melt). (ii) In the presence of the TB detection probe signal (*C*_*T*_ ≤ 37) and no more than one *rpoB* SMB-positive signal (*C*_*T*_ ≤ 40) along with the presence or absence of the IC signal, the result was “MTB trace detected but RIF resistance indeterminate” (the assay did not proceed to melt). (iii) In the presence of a TB signal plus ≥2 *rpoB* SMB signals, the assay was triggered to proceed to the post-PCR melt stage, and based on the *rpoB* probe *C*_*T*_s and the *T*_*m*_ values obtained, the following calls were made: “MTB detected high/medium/low/very low” and “RIF resistance detected/not detected," respectively (if <4 *rpoB* SMB probes generated *T*_*m*_ values under these circumstances, the output was changed to “RIF resistance indeterminate”). The distinct separation of the assay into the real-time amplification and post-PCR melt stages allowed the identification of TB-negative and “trace” TB samples within approximately 1 h and RIF susceptibility calls in less than 90 min. A full description of the assay parameters is provided in the supplemental material and Table S4.

10.1128/mBio.00812-17.5 FIG S4  Ultra assay *M. tuberculosis* and RIF resistance detection algorithm. Flow chart depicting the algorithm used for the detection of *M. tuberculosis* and the presence of RIF resistance in a clinical sample. Download FIG S4, EPS file, 1.3 MB. Copyright © 2017 Chakravorty et al. 2017 Chakravorty et al. This content is distributed under the terms of the Creative Commons Attribution 4.0 International license.

10.1128/mBio.00812-17.9 TABLE S4  Ultra assay cycling parameters. Download TABLE S4, DOCX file, 0.01 MB. Copyright © 2017 Chakravorty et al. 2017 Chakravorty et al. This content is distributed under the terms of the Creative Commons Attribution 4.0 International license.

### Analytical sensitivity testing of Ultra in comparison with Xpert.

The analytical sensitivity and limit of detection (LOD) of the extremely drug-resistant-TB assay was determined by spiking *M. bovis* BCG and an attenuated strain of *M. tuberculosis* H37Rv (mc^2^6030) into *M. tuberculosis*-negative sputum and testing each sample according to a standard protocol as described in Text S1. Analytical sensitivity experiments were performed by spiking known numbers of CFU in sputum in a specific dilution series from 10^7^ CFU/ml to 0 CFU/ml and performing the assay using a defined number of replicates (usually 20 per dilution). Xpert was performed on the same spiked sputum aliquots simultaneously with Ultra. The assay LOD was defined as the lowest number of CFU which, when spiked into 1 ml of sputum, would result in the detection of *M. tuberculosis* ≥95% of the time that a test was performed. The dynamic range of the assay was evaluated by performing similar experiments with sputa spiked with numbers of *M. tuberculosis* CFU ranging from 10^7^ to 1/ml in multiple replicates, and the semiquantitative prediction capacities of Xpert and Ultra were then calculated and compared. A detailed description of CFU stock preparation and sputum spiking protocols are included in Text S1.

10.1128/mBio.00812-17.1 TEXT S1  Description of cartridge composition, testing, and preparation of CFU and detailed PCR cycling parameters and references. Download TEXT S1, DOCX file, 0.03 MB. Copyright © 2017 Chakravorty et al. 2017 Chakravorty et al. This content is distributed under the terms of the Creative Commons Attribution 4.0 International license.

### Mutation panel challenge, hetero-resistance detection, and inclusivity testing.

The mutation panel challenge was performed using *M. tuberculosis* DNA provided by the Foundation of Innovative New Diagnostics (FIND) as well as DNA samples from clinical TB isolates maintained at Rutgers University. The DNA samples were isolated from a wide range of RIF-R clinical strains and confirmed by Sanger sequencing to contain a variety of mutations across the *rpoB* RRDR (Table 1). The Ultra cartridge was preloaded with the DNA of interest, the loaded cartridge placed into the GeneXpert instrument bay, and the assay performed by selecting a version of an automated assay protocol that was slightly modified to permit testing of DNA rather than CFU. The different wild-type and mutant DNA samples were run over a period of 6 months in 32 different modules in the GeneXpert instrument using different cartridge lots to check for *T*_*m*_ reproducibility. To test for the capacity of the assay to detect hetero-resistance, mutant DNA was added to wild-type DNA at various percentages, and the mixed DNA samples were run using the Ultra cartridges to evaluate the smallest amount of mutant DNA necessary in the mixed sample to trigger a RIF-R call. Inclusivity testing was performed on at least four replicates of DNA isolated from 25 different *M. tuberculosis* and *M. bovis* strains with various copies of IS*6110*, ranging from 0 copies to as many as many as 18 copies representing various genotypic lineages (Table S1). Appropriate positive (quantified *M. tuberculosis* DNA) and negative (PCR-grade water) controls were also run in multiple replicates with all the experiments.

### Evaluation of analytical specificity and exclusivity.

The analytical specificity and exclusivity of Ultra were tested using concentrated saturated bacterial liquid cultures (approximately 10^7^ to 10^8^ CFU/ml) of 30 different species of nontuberculous mycobacteria (NTM) and common bacterial microflora present in sputum and the upper respiratory tract. In the case of the Gram-positive and -negative bacteria for which culture was not available, 10^8^ genome equivalents of DNA were used instead of the culture. The NTM species included a laboratory strain of *Mycobacterium smegmatis* and several strains each of 29 different NTM isolates obtained from the ATCC repository (Manassas, VA) and National Jewish Health (Denver, CO), consisting of the following *Mycobacterium* species: *M. abscessus*, *M. asiaticum*, *M. avium*, *M. celatum*, *M. chelonae*, *M. flavescens*, *M. fortuitum*, *M. gastri*, *M. genavense*, *M. gordonae*, *M. goodii*, *M. haemophilum*, *M. interjectum*, *M. intracellulare*, *M. kansasii*, *M. marinum*, *M. malmoense*, *M. mucogenicum*, *M. peregrinum*, *M. phlei*, *M. scrofulaceum*, *M. shimoidei*, *M. simiae*, *M. szulgai*, *M. terrae*, *M. thermoresistibile*, *M. triviale*, *M. xenopi*, and *M. vaccae*. The Gram-positive and the Gram-negative bacteria included *Streptococcus agalactiae*, *Streptococcus pneumoniae*, *Streptococcus mitis*, *Streptococcus mutans*, *Streptococcus pyogenes*, *Staphylococcus aureus*, *Staphylococcus epidermidis*, *Haemophilus influenzae*, *Neisseria* sp., *Nocardia* sp., *Corynebacterium* sp., *Escherichia coli*, *Pseudomonas aeruginosa*, *Klebsiella pneumoniae*, *Acinetobacter baumannii*, *Citrobacter freundii*, *Moraxella catarrhalis*, and *Enterobacter cloacae*. The Gram-positive and Gram-negative bacteria were not incubated in sample reagent for more than 2 min to prevent lysis prior to loading in the sample chamber. The NTM were incubated for the usual 15 min, as was done for *M. tuberculosis*. To check for the ability of Ultra to correctly detect *M. tuberculosis* against a background of NTM DNA, 10^6^ genome equivalents of NTM DNA were mixed with 50 genome equivalents of *M. tuberculosis* DNA and tested for TB detection and RIF-R calls. All the assays were performed using the Ultra cartridges as described above.

### Retrospective accuracy study on clinical samples.

The performance of Ultra on clinical sputum samples was done in a limited retrospective accuracy study at the National Reference Laboratories in Belarus and Georgia and at the P. D. Hinduja Hospital and Medical Research Center in Mumbai, India. The sputum samples used in the study consisted of two sets of well-characterized deidentified frozen samples from study participants presenting with symptoms compatible with TB. The first set (212 samples) came from the FIND specimen bank bio-repository, representing different geographical locations (Peru, Vietnam, South Africa), and the second set (65 samples) was prospectively collected at two of the sites (Georgia and India). Two sputum samples were characterized per study participant within 24 h of sputum collection, and testing included two sets of smear microscopy (either Ziehl-Neelsen or auramine O staining) and solid (Löwenstein Jensen media) and liquid (mycobacterial growth indicator tubes [MGIT]; Becton, Dickinson, Sparks, MD) cultures of each sample, followed by species confirmation for positive cultures (MPT64 antigen detection). RIF susceptibility was determined by either the proportion method on Löwenstein Jensen medium or the MGIT SIRE kit (Becton, Dickinson, Sparks, MD). Xpert was performed with 1 ml of fresh sputum sample, and Ultra was later performed on an approximately 1-ml frozen aliquot of the same sputum sample according to the manufacturer’s instructions (Cepheid, Inc., Sunnyvale, CA). The performances of Ultra and Xpert were evaluated against culture and RIF susceptibility testing as the gold standard.

### Statistical analysis.

For calculation of the LOD values, the percentages of the replicates resulting in successful TB detection and RIF susceptibility calls were calculated at each input CFU concentration in sputum for both Ultra and Xpert. Binary logistic regression results were fitted through the tested concentrations, and lower and upper 95% confidence intervals (95% CIs) were generated for the curve. The 95% CI for the minimum input concentration was determined by where the 95% probability level crossed the upper and lower 95% CIs, which indicated the LOD. Sensitivity and specificity values in the clinical accuracy study were calculated at the 95% CI for Ultra and Xpert.
