# Supplementary material for: The New Xpert MTB/RIF Ultra: Improving Detection of Mycobacterium tuberculosis and Resistance to Rifampin in an Assay Suitable for Point-of-Care Testing
Source: mBio. 2017 Aug 29;8(4):e00812-17. doi: 10.1128/mBio.00812-17 (PMC5574709; doi:10.1128/mBio.00812-17)
Supplement: TABLE S1 [file mbo004173453st1.docx]

**Supplementary Table 1**: Inclusivity testing results for the Ultra assay

| **Strain** | **IS6110 copies** | **MTB detected** |
| --- | --- | --- |
| Clinical | 0 | Yes |
| Clinical | 1 | Yes |
| Clinical | 5 | Yes |
| Clinical | 4 | Yes |
| Clinical | 13 | Yes |
| Clinical | 10-12 | Yes |
| Clinical | 18 | Yes |
| Clinical | 15 | Yes |
| Clinical | 10 | Yes |
| Clinical | 8 | Yes |
| Clinical | 8 | Yes |
| Clinical | 3 | Yes |
| Clinical | 2 | Yes |
| Clinical | 3 | Yes |
| Clinical | 4 | Yes |
| Clinical | 10-12 | Yes |
| Clinical | 10 | Yes |
| Clinical | 11 | Yes |
| Clinical | 8-10 | Yes |
| Clinical | 12 | Yes |
| *M. tuberculosis* H37Rv | 16 | Yes |
| *M. bovis* BCG | 1 | Yes |
| *M. bovis* | 1 | Yes |
