# Supplementary material for: The New Xpert MTB/RIF Ultra: Improving Detection of Mycobacterium tuberculosis and Resistance to Rifampin in an Assay Suitable for Point-of-Care Testing
Source: mBio. 2017 Aug 29;8(4):e00812-17. doi: 10.1128/mBio.00812-17 (PMC5574709; doi:10.1128/mBio.00812-17)
Supplement: TABLE S2 [file mbo004173453st2.docx]

**Supplementary Table 2**: Sequences of the primers used in the Ultra assay

| **PCR** | **Target gene** | **Primer name** | **Sequence (5’-3’)** |
| --- | --- | --- | --- |
| **Phase 1** | Internal Control | BG-F | AGCGCTTGCGGCAAA CACGGAGAAA |
|  |  | BG-R1 | GCGACACCGGCGAATACAGAGATACC |
|  | *rpoB* | rpoB-F1 | CGTGGAGGCGATCACACCGCAGAC |
|  |  | rpoB-R | AGCTCCAGCCCGGCACGCTCACGT |
|  | *IS6110* | IS6110-OF | AGCGCCGCTTCGGACCACCAG |
|  |  | IS6110-OR | AGGCGTCGGTGACAAAGGCCACGTA |
|  | *IS1081* | IS1081-OF | CAGCCCGACGCCGAATCAGTTGTT |
|  |  | IS1081-OR | GGTGCGGGCGGTGTCGAGGTG |
| **Phase 2** | Internal Control | BG-F | AGCGCTTGCGGCAAA CACGGAGAAA |
|  |  | BG-R2 | GCTCCACCGAACAATCCGATCTTACCGCCC |
|  | *rpoB* | rpoB-F2 | ATCAACATCCGGCCGGTGGTCGCC |
|  |  | rpoB-R2 | TCACGTGACAGACCGCCGGGC |
|  | *IS6110* | IS6110-I-F | CGCCGCTTCGGACCACCAGCAC |
|  |  | IS6110-I-R | GTGACAAAGGCCACGTAGGCGAACC |
|  | *IS1081* | IS1081-I-F | GCGCGGCAAGATCATCAATGTGGAG |
|  |  | IS1081-I-R | GCCACCGCGGGGAGTTTGTCG |

F: Forward, R: Reverse, O: Outer, I: Inner. All the other letters represent nucleotides
