# Supplementary material for: The New Xpert MTB/RIF Ultra: Improving Detection of Mycobacterium tuberculosis and Resistance to Rifampin in an Assay Suitable for Point-of-Care Testing
Source: mBio. 2017 Aug 29;8(4):e00812-17. doi: 10.1128/mBio.00812-17 (PMC5574709; doi:10.1128/mBio.00812-17)
Supplement: TABLE S3 [file mbo004173453st3.docx]

**Supplementary Table 3**: Sequences of the probes used in the Ultra assay

| **Target gene** | **Probe name** | **Probe type** | **Dye** | **Sequence (5’-3’)** |
| --- | --- | --- | --- | --- |
| Internal control | BG-MB | MB | CF1 | CggccgaCAGACAAGCTCCGTCATTTGAtcggcc |
| *rpoB* | Rpo1-SMB | SMB | CF5 | cgaccgCccatAaattggctcagctggctggtgAcggtcg |
|  | Rpo2-SMB | SMB | CF4 | ggcgcgaaccAcgacagcgggttgttctggtccatgaacgcgcc |
|  | Rpo3-SMB | SMB | CF6 | cgcggccgacagtTggcgcttgtgggtTaaccccgacgccgcg |
|  | Rpo4-SMB | SMB | CF3 | cgcgcgccgggccccagcAccAacagtcggAgcttcgcgcg |
| *IS1081* | IS1081-MB | MB | FAM | cgcagcGGTACTCGACGCTCTGACCGACAAgctgcg |
| *IS6110* | IS6110-TM | TM | FAM | CGGCTGTGGGTAGCAGACCTCACC |

The lowercase letters represent the stem portion and the uppercase letters represent the probe portion of the *rpoB* SMBs and the BG and *IS1081* MB. CF represents proprietary fluorescent dyes manufactured by Cepheid Inc. (Sunnyvale, CA, USA). All the probes have the dyes attached at the 5’ end and an appropriate quencher at the 3’ end except for the BG MB which has the quencher attached to an internal nucleotide near the 3’ end. The BG-MB consisted of an extra Cytosine residue at the 5’ end of the MB probe due to design requirements. MB: Molecular Beacon, SMB: Sloppy Molecular Beacon, TM: Taqman probes
