# Supplementary material for: The New Xpert MTB/RIF Ultra: Improving Detection of Mycobacterium tuberculosis and Resistance to Rifampin in an Assay Suitable for Point-of-Care Testing
Source: mBio. 2017 Aug 29;8(4):e00812-17. doi: 10.1128/mBio.00812-17 (PMC5574709; doi:10.1128/mBio.00812-17)
Supplement: TABLE S4 [file mbo004173453st4.docx]

| **Stage** | **No. of cycles** | **Temperature (^0^C)** | **Time (sec)** | **Optics** |
| --- | --- | --- | --- | --- |
| **Phase 1** | 31 | 97 | 3 | Off |
|  |  | 72 | 20 | Off |
| **Phase 2** | 15 | 64 | 5 | Off |
|  |  | 64 | 5 | On |
|  | 24 | 97 | 3 | Off |
|  |  | 64 | 10 | On |
|  |  | 74 | 10 | Off |
|  | 1 | 97 | 3 | Off |
|  |  | 64 | 10 | On |
|  |  | 74 | 10 | Off |
|  | Hold | 74 | 2 | Off |
|  | **Decision for melt** | | |  |
| **Melt** | 5 | 95 | 5 | Off |
|  |  | 68 | 20 | Off |
|  | Hold | 95 | 120 | Off |
|  | Hold | 50 | 10 | Off |
|  | Melt Curve | 50-85 | continuous | On |

**Supplementary Table 4**: Ultra assay cycling parameters
