# Supplementary material for: The New Xpert MTB/RIF Ultra: Improving Detection of Mycobacterium tuberculosis and Resistance to Rifampin in an Assay Suitable for Point-of-Care Testing
Source: mBio. 2017 Aug 29;8(4):e00812-17. doi: 10.1128/mBio.00812-17 (PMC5574709; doi:10.1128/mBio.00812-17)
Supplement: TEXT S1 [file mbo004173453s1.docx]

**Supplementary methods:**

**Cartridge configuration, assay composition and testing procedure.** The Ultra assay used a modified version of the filter-based sample processing/PCR Cartridge A (Cepheid, Sunnyvale CA) as was developed for the Xpert MTB/RIF assay (1). The essential components of the cartridge were similar to the Xpert Cartridge, including the presence of an integrated PCR tube, a multi-position fluidic valve, a bacterial-capture filter, and 11 chambers that contained all the buffers necessary for sample processing and PCR. However, the integrated PCR tube used in the Ultra assay had a 50µl capacity instead of the 25µl capacity PCR tube used in the Xpert assay. Lyophilized reagent beads used for the two phase amplification in the assay were placed into two of the cartridge chambers. The first set of beads contained reagents necessary for amplification of the *M. tuberculosis* detection targets *IS6110*, and *IS1081*, RIF resistance detection target *rpoB* and an internal control sequence of *Bacillus globigii* (a 4-plex PCR amplification). The second set of beads contained reagents necessary to perform nested or hemi-nested PCR of the first set of amplicons, 1 molecular beacon probe for detecting the *IS1081* and 1 Taqman probe for detecting the *IS6110* amplicons labeled with a single fluorophore, 1 molecular beacon for detection of the internal control amplicon and 4 different Sloppy Molecular Beacon probes for detecting the RIF resistance inducing mutations in the *rpoB* RRDR region.

To perform a test, each sample (spiked sputum, patient samples, cultured *M. tuberculosis* or *M. bovis* BCG CFU) was first mixed at a 2:1 ratio with an NaOH and isopropanol containing sample reagent (SR) as described previously (1); the sample was then added to the sample loading chamber of the cartridge. The loaded cartridge was placed into a GeneXpert instrument running software developed for the Ultra assay (Cepheid, Sunnyvale, CA). The assay was then started and automated processing of the sample for DNA isolation followed by the two-phase PCR assay and melt analysis was performed. Briefly, the lyophilized *B. globigii* spores were mixed with the sample, and the sample was then filtered through a bacterial capture filter within the cartridge to capture the *M. tuberculosis* bacilli and *B. globigii* spores present in the sample. The captured bacteria were then washed on the cartridge filter several times with a wash buffer, and the *M. tuberculosis* and *B. globigii* DNA released by highly efficient lysis through sonication. The resulting DNA solution was used to resuspend the first set of PCR reagent beads and then this PCR mix was aspirated into the integrated PCR tube for a first round of amplification. After this first PCR, a specific amount of the amplified sample was moved out of the tube to waste, and the PCR tube was then filled with a second set of PCR reagent beads that had been resuspended in buffer and mixed with the remaining amplicon in the tube. A second PCR was then performed followed by a post PCR melt analysis, depending on the amplification signals obtained in the second PCR. The Tm values were identified by the automated GeneXpert Tm calling software (Cepheid, Sunnyvale, CA) and classified as Tm values that identified wild type or mutant *rpoB* amplicon sequence based on pre-defined Tm parameters (Tm windows). These values were then used to determine whether or not a RIF susceptibility call was made.

**Composition of the PCR beads and the PCR assay parameters**. Lyophilized PCR beads were hydrated and solubilized during the automated PCR protocol and delivered into the PCR tube attached to the cartridge employing microfluidics that allowed a two phase nested (for *rpoB*, *IS6110* and *IS1081* assays) and hemi-nested PCR (for the internal control assay). Phase 1 PCR was performed using the *M. tuberculosis* DNA isolated from the sample inside the cartridge and the phase 2 assay was performed using the amplicons generated in the phase 1 PCR. Each PCR reagent bead contained lyophilized PCR reagents including Taq Polymerase Enzyme, MgCl_2_, dNTPs, proprietary lyophilization buffer components and the primers and probes (Supplementary tables 2 and 3) used in the respective PCR phases. The phase 1 reagent beads contained 0.25 to 0.4µM of four different primer pairs (Supplementary table 2), 2.7mM MgCl_2_ and 400µM of dNTPs; the phase 2 bead contained 1µM to 0.25 µM of the four nested or hemi-nested primer pairs (Supplementary table 2) and 4mM MgCl_2_ and same amount of dNTPs as the phase 1 bead and approximately 150-800 nM of probes (Supplementary table 3). The cycling parameters of the Ultra assay which consisted of two different amplification phases followed by a post PCR melt curve based on the signals obtained in the phase 2 PCR, are summarized in supplementary table 4.

**Preparation of mixed DNA to test for detection of heteroresistance**. The *rpoB* RRDR of DNA samples obtained from different clinical strains were confirmed by Sanger sequencing for the presence or the absence of mutations and quantified by using Nanodrop Microvolume Spectrophotometer (Thermo Fisher Scientific, Wilmington, DE, USA). Mixtures of wild type DNA and DNA containing the different mutations were prepared by mixing the same concentration of both DNA at different percentages, from 90% mutant DNA to 0.5% mutant DNA in the mixture. The Ultra assay cartridge was pre-loaded with the DNA mixture, the loaded cartridge placed into the GeneXpert instrument bay and the assay performed by selecting a version of an automated assay protocol that was slightly modified to permit testing of DNA rather than *M. tuberculosis* CFU.

**Preparation of *M. tuberculosis* and *M. bovis* culture stocks and determination of CFU.** Attenuated strains of *M. tuberculosis* H37Rv (mc^2^6030), a kind gift of William R Jacobs Jr., Albert Einstein College of Medicine, and *M. bovis* BCG were cultured by inoculating an optical density 600 nm (OD^600^) culture 1:100 in 10 ml of 7H9 broth supplemented with 10% Middlebrook OADC Growth supplement and 0.05% Tween 80 (Sigma Aldrich, St Louis, MO). *M. tuberculosis* H37Rv (mc^2^6030) has independent deletions in the *panC* and *panD* genes and requires media supplemented with 24 µg/ml of Calcium Pantheonate (Sigma Aldrich, St Louis, MO) (2). Both of the strains were then grown to an optical density OD^600^ of 0.6-0.8 and sub-cultured one additional time by making a 1 to 100 dilution into fresh growth media and re-incubating the culture until the OD^600^ again reached a density of 0.6-0.8. This final culture stock was used for the CFU platting, dilutions, and aliquoting described below. First, the culture stock was mixed three times using a serological pipette, then divided into 200µl aliquots and stored at -80^o^C until use. An additional 1 ml aliquot of the stock culture was sonicated for 30 seconds using a Branson CPX1800 Ultrasonic water-bath (Branson, Danbury, CT, USA) and rested for 30 sec on ice. This was repeated twice more and then the culture was placed on ice for six min. Tenfold serial dilutions were then performed by adding 400 µl of the sonicated culture to 3.6 ml of supplemented media; subsequent dilutions were performed in the same manner except that each dilution was mixed by pipetting ten times, aspirating from the bottom of the tube and releasing on the top, and then ten times aspirating from the top and releasing on the bottom. The 10^-5^, 10^-6^ and 10^-7^ dilutions were then plated in triplicate on 7H10 agar plates supplemented with 10% Middlebrook OADC Growth supplement (and 24 µg/ml Calcium Pantheonate for the H37Rv mc^2^6030 strain only). Culture plates were checked for growth at one week after plating and then on alternate days after that. Colony counts were performed once colonies were clearly visible (at approximately two to three weeks). Colony counts ranging between 300-10 were included for estimation of the CFU/ml in the respective cultures. Plates with CFU >300 (too numerous to count) or <10 (unreliable CFU numbers) were excluded to ensure most accurate estimation of the CFU/ml in the prepared culture stock.

**Dilutions and spiking in sputum for analytical studies:** A frozen 200 µl aliquot of *M. tuberculosis* H37Rv (mc^2^6030) or *M. bovis* BCG culture stock (as mentioned above) was allowed to thaw in ice, and 800 µl of supplemented 7H9 media was added to the vial. The vial was vortexed for 30 seconds at medium speed and placed on ice for 6 minutes to allow any aerosolized particles to settle. The vial was then sonicated and serial dilutions performed as described above for CFU plating, except that in addition to ten-fold dilutions, five-fold or two-fold serial dilutions were also performed in 2-4 ml of supplemented 7H9 media as needed to produce solutions containing between 10^7^ CFU/ml and 500 CFU/ml, depending on the experimental requirements. The remaining sonicated stock, if used for subsequent dilution series was stored at 4^0^C for no longer than 7 days. When the stored vial was used for spiking studies on subsequent days, the stock suspension was sonicated once for 30 seconds. If used on additional days, the sample was vortexed at medium setting for 2 minutes for a maximum of one such vortex every 24 hours. For spiked sputum studies, sputa were vortexed briefly and allowed to rest for 5-6 minutes before using in analytical experiments.

**References:**

1. Helb D, Jones M, Story E, Boehme C, Wallace E, Ho K, Kop J, Owens MR, Rodgers R, Banada P, Safi H, Blakemore R, Lan NT, Jones-Lopez EC, Levi M, Burday M, Ayakaka I, Mugerwa RD, McMillan B, Winn-Deen E, Christel L, Dailey P, Perkins MD, Persing DH, Alland D. 2010. Rapid detection of Mycobacterium tuberculosis and rifampin resistance by use of on-demand, near-patient technology. J Clin Microbiol 48:229-37.

2. Sambandamurthy VK, Wang X, Chen B, Russell RG, Derrick S, Collins FM, Morris SL, Jacobs WR, Jr. 2002. A pantothenate auxotroph of Mycobacterium tuberculosis is highly attenuated and protects mice against tuberculosis. Nat Med 8:1171-4.
